# Special Issue “Advances in Molecular Forensic Pathology and Toxicology: An Update”

**DOI:** 10.3390/ijms27146382

**Published:** 2026-07-17

**Authors:** Angelo Montana

**Affiliations:** Department of Excellence Biomedical Sciences and Public Health, Marche Polytechnic University, 60121 Ancona, Italy; a.montana@univp.it

## Introduction

Forensic pathology and forensic toxicology are experiencing one of the most significant scientific transformations since the introduction of molecular biology into biomedical research. Traditionally, forensic investigations have relied on the integration of autopsy findings, histopathology, toxicological analyses, and circumstantial evidence to reconstruct the cause, mechanism, and timing of death [1,2,3]. Although these approaches remain the cornerstone of forensic practice, they are frequently limited by postmortem degradation, environmental influences, biological variability, and the intrinsic complexity of many medico-legal cases [4,5,6].

Recent advances in molecular sciences are progressively overcoming these limitations. High-throughput technologies—including transcriptomics, metabolomics, epigenomics, microbiomics, and systems biology—have opened new opportunities for investigating postmortem biological processes at an unprecedented level of resolution. Rather than focusing exclusively on morphological alterations or the detection of xenobiotics, modern forensic medicine is increasingly oriented toward identifying dynamic molecular signatures capable of reconstructing the biological events occurring before, during, and after death. This paradigm shift is paving the way toward what may be defined as precision forensic medicine, in which molecular biomarkers complement conventional forensic investigations to improve diagnostic accuracy, postmortem interval estimation, and the interpretation of complex causes of death [7,8].

The contributions collected in this Special Issue reflect this ongoing evolution by presenting innovative approaches that span molecular pathology, forensic toxicology, biomarker discovery, and multi-omics technologies. Although addressing different forensic questions, these studies collectively highlight a common objective: translating molecular research into practical tools capable of supporting forensic decision-making.

One of the most active research areas represented in this Special Issue concerns the molecular estimation of the postmortem interval (PMI), which remains one of the greatest unresolved challenges in forensic pathology. Traditional methods—including body cooling, livor mortis, rigor mortis, vitreous chemistry, and forensic entomology—are all influenced by numerous intrinsic and environmental variables that reduce their accuracy, particularly during the early postmortem period. Consequently, increasing attention has been devoted to identifying molecular biomarkers characterized by predictable postmortem kinetics.

In this context, the systematic review by Cianci et al. [9] offers a critical appraisal of the current evidence on microRNAs as markers for PMI estimation, emphasizing both their remarkable postmortem stability and the obstacles that still hinder their routine forensic implementation. The review highlights the usefulness of miRNAs both for PMI estimation and for normalization purposes, largely by virtue of that stability. This behavior, however, is far from uniform: some miRNAs remain particularly stable over long intervals and across different tissues, while others degrade more rapidly. Several further variables can influence these molecules, among which the type of tissue, the cause of death, and circadian rhythm appear the most relevant. Although the available results are encouraging, the evidence remains limited and fragmented, and further research is needed before these findings can be considered solid and transferable across settings.

Complementing this comprehensive overview, the experimental study by Fu et al. [10] explores the forensic potential of circular RNAs, a still under-investigated class of highly stable non-coding RNAs, by testing their correlation with PMI. The authors worked on liver tissue from mice, exposed it to four temperatures (4, 18, 25, and 35 °C), and followed the behavior of circRnf169 at six defined time points after death, using 28S rRNA as a reference gene. What emerged was that at 4 °C, the transcript decreased at a steady rate, whereas at the other temperatures it collapsed so abruptly that no information on PMI could be retrieved. The authors therefore conclude by proposing a mathematical model to estimate early PMI, while calling for the identification of additional markers in animal models and validation in human samples.

Similarly, Choi et al. [11] investigated postmortem mRNA degradation, correlating it with the morphological changes followed in parallel on histological preparations of brain and skeletal muscle stained with hematoxylin–eosin. Sprague–Dawley rats were examined starting immediately after death and followed for 21 days, with repeated assessments at defined intervals, each conducted under two storage conditions: 4 °C and 26 °C. As regards histology, the samples kept at 4 °C were still readable at 21 days, whereas at 26 °C, cell destruction was visually confirmed after 14 days. As regards RNA, total RNA was extracted from each sample and reverse-transcribed, and three housekeeping genes, namely Gapdh, Sort1, and B2m, together with 5S ribosomal RNA, were quantified by RT-qPCR using a SYBR Green assay. The targets diverged sharply: Gapdh and 5S rRNA held up well over time and thus stand out as the preferable RNA targets for PMI estimation in these tissues, whereas Sort1 and B2m combined poor stability with low expression levels.

Read alongside the previous two, this study shows forensic pathology gradually shifting from the exclusive observation of postmortem physical change toward a quantitative reading of the molecular events that underlie it. What is emerging is not the search for one ideal biomarker, but the construction of integrated molecular panels, in which several markers are combined precisely because their complementarity is what improves accuracy and reproducibility.

The evolution toward molecular investigation is equally evident in forensic toxicology. Traditionally centered on the identification and quantification of drugs and toxic compounds, forensic toxicology is increasingly incorporating biomarkers that reflect the biological response induced by toxic exposure. This functional perspective allows investigators to better characterize intoxication mechanisms, distinguish acute from chronic exposure, and improve the interpretation of toxicological findings [12,13,14,15].

Within this framework, Ptaszyńska-Sarosiek et al. [16] investigated whether systemic markers of oxidative stress, namely total antioxidant capacity (TAC), total oxidative status (TOS), and the oxidative stress index (OSI), can serve as indirect indicators of acute alcohol intoxication rather than chronic dependence. Redox status was assessed in both blood and urine across three groups: 105 sudden deaths used as controls, 47 deaths from alcohol overdose, and 102 individuals with alcohol dependence. Blood proved the more informative matrix, with TAC emerging as a plausible marker of alcohol consumption. The authors interpret the rise of TAC over TOS as an adaptive response to the overproduction of reactive oxygen species induced by alcohol exposure. Their findings confirm a disruption of redox homeostasis in chronic alcohol use and support TAC, TOS, and OSI as usable indicators of exposure.

Perhaps the most forward-looking contribution of this Special Issue integrates several molecular layers into a single investigative framework. Bai et al. [16] couple untargeted metabolomics with gut microbiome profiling to separate fatal anaphylaxis from other forms of sudden death. In rat models (*n* = 12) reproducing fatal anaphylaxis, acute myocardial infarction, and coronary atherosclerosis with anaphylaxis, plasma metabolomics and 16S rRNA sequencing of fecal matter, processed through a Random forest classifier, yielded three candidate metabolites (tryptophan, trans-3-indoleacrylic acid, and imidazole acetic acid) and three bacterial genera; the microbial signature outperformed serum IgE without matching serum tryptase. The decisive step is the test on real casework: when the metabolomic component alone was applied to forensic samples from anaphylactic and non-anaphylactic deaths (*n* = 12), tryptophan proved the most resilient of the three metabolites in cadaveric blood, with a diagnostic performance (AUC = 87.1528) exceeding that of both IgE and tryptase. It is this finding that makes it, in the authors’ view, the most promising postmortem biomarker of fatal anaphylaxis.

A further contribution to this convergence comes from artificial intelligence (AI), whose applications in thanatology are being developed, particularly for postmortem identification [17], the estimation of the postmortem interval [18], and the determination of the causes of death [19,20].

Taken together, these contributions document a broader transformation of the forensic sciences: from a descriptive discipline toward a data-driven molecular one, in which transcriptomics, metabolomics, microbiomics, oxidative stress biology, and computational analysis converge to reconstruct postmortem biological processes with increasing precision. This evolution does not replace traditional forensic pathology; it complements and strengthens it, supplying objective molecular evidence in support of medico-legal conclusions.

Future developments will likely be driven by the combination of these technologies with spatial transcriptomics, single-cell sequencing, machine learning, and advanced bioinformatics, from which multidimensional molecular fingerprints may emerge, capable of refining cause-of-death determination, postmortem interval estimation, forensic toxicology, and the molecular autopsy. Substantial challenges nonetheless remain: biomarker standardization, multicenter validation, reproducibility, ethical implications, and the translation of research findings into routine forensic practice.

In conclusion, the articles collected in this Special Issue provide compelling evidence that molecular forensic pathology and toxicology are entering a new era. The integration of innovative biomarkers with advanced analytical technologies is progressively reshaping forensic investigations, bringing the field closer to the vision of precision forensic medicine. Continued interdisciplinary collaboration among forensic pathologists, molecular biologists, toxicologists, bioinformaticians, and clinicians will be essential to fully realize the diagnostic and translational potential of these emerging molecular approaches.
